# Role of Vitamin K-Dependent Factors Protein S and GAS6 and TAM Receptors in SARS-CoV-2 Infection and COVID-19-Associated Immunothrombosis

**DOI:** 10.3390/cells9102186

**Published:** 2020-09-28

**Authors:** Anna Tutusaus, Montserrat Marí, José T. Ortiz-Pérez, Gerry A. F. Nicolaes, Albert Morales, Pablo García de Frutos

**Affiliations:** 1 Department of Cell Death and Proliferation, IIBB-CSIC, IDIBAPS, 08036 Barcelona, Spain; anna.tutusaus@iibb.csic.es (A.T.); monmari@clinic.cat (M.M.); 2 Clinic Cardiovascular Institute, Hospital Clinic Barcelona, 08036 Barcelona, Spain; JTORTIZ@clinic.cat; 3 Centro de Investigación Biomédica en Red sobre Enfermedades Cardiovasculares (CIBERCV), 28029 Madrid, Spain; 4 Department of Biochemistry, Cardiovascular Research Institute Maastricht (CARIM), Maastricht University, 6200 MD Maastricht, The Netherlands; g.nicolaes@maastrichtuniversity.nl; 5 Barcelona Clinic Liver Cancer (BCLC) Group, Liver Unit, Hospital Clínic, CIBEREHD, 08036 Barcelona, Spain

**Keywords:** AXL, MERTK, GAS6, viral infection, coagulation, endothelium, immune response

## Abstract

The vitamin K-dependent factors protein S (PROS1) and growth-arrest-specific gene 6 (GAS6) and their tyrosine kinase receptors TYRO3, AXL, and MERTK, the TAM subfamily of receptor tyrosine kinases (RTK), are key regulators of inflammation and vascular response to damage. TAM signaling, which has largely studied in the immune system and in cancer, has been involved in coagulation-related pathologies. Because of these established biological functions, the GAS6-PROS1/TAM system is postulated to play an important role in SARS-CoV-2 infection and progression complications. The participation of the TAM system in vascular function and pathology has been previously reported. However, in the context of COVID-19, the role of TAMs could provide new clues in virus-host interplay with important consequences in the way that we understand this pathology. From the viral mimicry used by SARS-CoV-2 to infect cells, to the immunothrombosis that is associated with respiratory failure in COVID-19 patients, TAM signaling seems to be involved at different stages of the disease. TAM targeting is becoming an interesting biomedical strategy, which is useful for COVID-19 treatment now, but also for other viral and inflammatory diseases in the future.

## 1. Introduction

TAM receptors (TYRO3, AXL, and MERTK) are a subfamily of receptor tyrosine kinases (RTKs). Being first cloned in 1991 based on RTK domain’s homology [1], no homolog proteins are found in *Caenorhabditis elegans* or *Drosophila melanogaster* as TAMs appear late in evolution [2]. Besides the intracellular tyrosine kinase domain, TAM receptors comprise a single-pass transmembrane domain and two immunoglobulin-like domains, followed by two fibronectin-type III repeats in the extracellular region. TAM receptors are widely expressed among human tissues, although their expression is noteworthy in immune cells (macrophages, monocytes, dendritic, and natural killer cells), platelets, endothelial cells, osteoclasts, Sertoli cells, as well as in the retinal pigment epithelium [3].

TAMs were orphan receptors until 1995, when Protein S (PROS1) and growth-arrest-specific 6 (GAS6) were identified as their ligands [4,5,6]. These proteins contain a C-terminal sex hormone-binding globulin (SHBG) domain, which is responsible for interaction with TAMs, preceded by four EGF-type domains in tandem and an N-terminal Gla-domain, found in vitamin K-dependent proteins (VKDPs) of the coagulation. The most remarkable feature of these ligands is their ability to bind phosphatidylserine (PtdSer), which is exposed on the cell surface of apoptotic cells and activated platelets. The γ-carboxylation of glutamate residues, a process that is dependent on vitamin K, enables the interaction of the Gla-domain with membrane-exposed PtdSer. Different specificities have been defined for TAM ligands: GAS6 binds all TAM receptors with higher affinity for AXL [7,8,9], while PROS1 principally engages MERTK and TYRO3, and only in specific cases has been suggested to activate AXL [10]. Ligand binding to Ig-like domains of TAM receptors triggers receptor dimerization, autophosphorylation of intracellular tyrosine, and signal transduction through different pathways, such as mitogen-activated protein kinase (MAPK), phosphoinositide 3-kinase–AKT (PI3K/AKT), or Janus kinase/signal transducers and activators of transcription (JAK/STAT) [11,12]. TAM heterodimers have been described [13,14] as well as TAM interaction with other receptors and their ligands [15,16], although the biological relevance of these alternative complexes is poorly understood. Similar to other RTKs, different mechanisms for TAM regulation have been postulated, such as an inhibitory phosphorylation site and dephosphorylation by tyrosine phosphatases. In addition, proteolytic cleavage of MERTK and AXL (and possibly TYRO3) by the disintegrin and metalloproteinase domain-containing protein 10 (ADAM10) and 17 (ADAM17) efficiently shuts down TAM activity [17,18].

## 2. TAM Receptor Functions

TAM receptor’s biological importance was made evident for the first time in the triple knock-out mice (TAM KO). Despite TAM KO mice being viable and apparently normal after birth, they become blind early in life and males are sterile, developing splenomegaly and an autoimmune lupus-like syndrome [19,20]. Since these initial studies, the contribution of TAM signaling in the regulation of the immune response and clearance of dead cells has been deciphered, as well as their prominent role in pathologies, such as cancer. The main TAM functions are briefly detailed below, as this topic has been reviewed previously [3,21,22,23,24].

### 2.1. Regulation of Immune Response

High levels of autoantibodies, skin lesions, joint swelling, and other features of autoimmune disease were described in TAM KO [20]. In addition, *Mertk−/−* were more susceptible to LPS-induced endotoxic shock [25] unveiling an immunomodulatory role of MERTK. Antigen presenting cells (APCs), mainly dendritic cells (DC) and macrophages, are indispensable for triggering inflammation and adaptive immunity. However, in order to avoid a disproportionate response, their activity must be rigorously controlled by different pathways, one of them being the TAM receptors. TAM-deficient APCs produce higher cytokine levels after toll like receptor (TLR) stimulation [15,25]. TAM activation inhibits inflammatory signaling downstream of TLR and cytokine receptors, such as nuclear factor-κB (NF-κB), MAPK, and tumor-necrosis factor (TNF) receptor-associated factor 3 and 6 (TRAF3/6), which results in a reduced release of certain cytokines [15]. This inhibition is mediated by the suppressor of cytokine signaling proteins, SOCS1 and SOCS3, which are upregulated after TAM activation. Moreover, Rothlin et al. described a cyclical regulation of APCs that involve AXL. After TLR stimulation, interferon receptor (INFAR) activation induced the expression of AXL. Then, AXL binds INFAR to switch its signaling from pro- to anti-inflammatory [15]. In addition, AXL may also regulate interferon (IFN) response through Twist, a transcriptional regulator of NF-κB, reducing TNF production [26]. TAMs also regulate inflammatory activity of natural killer cells by reducing IFNγ production and their proliferation through E3 ubiquitin ligase Cbl-b [27,28].

A consequence of these anti-inflammatory functions of the GAS6-PROS1/TAM system is its effect on models of acute inflammation. In particular, GAS6 has been shown to attenuate the ischemia reperfusion damage in liver [29], kidney [30], and lung [31]. Not surprisingly, the administration of recombinant GAS6 diminishes deleterious effects of sepsis, including acute lung injury [32] and the endothelial hyper-permeability that is associated with bacterial endotoxemia [33]. In this context, PROS1 could also play an important role in reprogramming macrophages to a reparative pro-resolving phenotype [34].

Nevertheless, inflammatory regulation by TAMs is not restricted to innate immunity [35]. A recent publication reported MERTK expression in T-cells after three days of TCR activation [36,37], in contrast to previous studies [38,39]. PROS1 stimulation of T-cells promoted the proliferation and cytokine secretion in a MERTK-dependent manner [36,37]. Moreover, local production of PROS1 by T-cells may represent a novel mechanism of crosstalk between the innate and the adaptive system. The eexpression of PROS1 in activated T-cells was first described in 1997 and later confirmed in both T-cell subsets, CD4^+^, and CD8^+^ [36,40,41]. T-cell regulated cytokine production in DC by PROS1/TAM interaction avoiding excessive immune response [41]. In the same line, PROS1 expression was upregulated in interleukin 4 (IL-4)-induced Th2, dampening DC activity via TYRO3 and limiting type 2 immunity [42].

### 2.2. Efferocytosis

Flip-flop of PtdSer during cell death enhances the binding of TAM ligands to apoptotic bodies. Subsequently, their binding to TAMs enables a closer interaction between apoptotic and phagocytic cells and the activation of cytoskeletal rearrangement, promoting clearance of apoptotic residues [43,44,45]. TAM signaling in this process, termed efferocytosis, is crucial for tissue homeostasis, as reflected in TAM KO mice. TAM deficiency frustrates proper testis development as Sertoli cells, which express all TAM receptors, fail to remove apoptotic spermatogenic cells [46,47]. Consequently, TAM KO males become infertile by three weeks of age [19]. Similarly, MERTK and TYRO3 regulate engulfment of photoreceptor distal membranes in the retinal pigment epithelium and their deficiency causes blindness in mice [48,49]. In humans, the mutations in MERTK gene are causative for certain cases of retinitis pigmentosa [50].

TAM signaling in APCs, particularly in macrophages, also contributes to debris clearance after homeostatic or pathological cell death. TAM deficiency reduces engulfment of apoptotic bodies without disturbing other phagocytic properties, indicative of an efferocytosis-specific TAM function [13,51]. Even if the role of MERTK in efferocytosis is more prominent, all of the TAM receptors participate in this process [13]. Individual TAM requirement observed in some studies might rely on cell and tissue specificity; for instance, MERTK seems to be induced by immunosuppressive and tolerogenic stimuli, while AXL prevailed upon inflammation [52]. Therefore, differences in expression pattern and ligand dependence are paramount in understanding the specific role of TAMs in homeostasis and disease development.

Interestingly, efferocytosis by APCs is associated with induction of T-cell anergy [53] mediated, in part, through TAM signaling. After pre-treatment with apoptotic cells, MERTK inhibits the NF-κB pathway, reducing the secretion of pro-inflammatory cytokines [54,55] and induces an immunosuppressive profile, characterized by high interleukin-10 (IL-10) and transforming growth factor-beta (TGFβ) and low type I IFN levels [56]. In fact, *Mertk−/−* mice displayed low levels of IL-10 and TGF-β after vesicular stomatitis virus (VSV) infection, resulting in abrogation of innate anergy, which is associated with enhanced VSV replication and poor survival after infection [56]. The translation of these results to infections with other viruses, such as SARS-CoV-2, is uncertain. Patients with severe COVID-19 show marked alterations in phenotypical and functional properties in T lymphocytes that may be used to counteract the excessive immunopathology that was observed in the lung [57]. Of note, dexamethasone, which has been approved as COVID-19 treatment for seriously ill patients, is a potent activator of the PROS1/MERTK axis [37]. An interesting aspect for analysis is if dexamethasone upregulation of MERTK and the consequent enhancing of innate anergy could play a positive role in COVID-19 [58].

Moreover, MERTK-mediated efferocytosis stimulated repair response through the activation of extracellular-signal-regulated kinase (ERK) and c-Jun N-terminal kinase (JNK) pathway via Ras homolog family member A (RhoA) and the production of hepatic growth factor [59]. These convergent functions may be implicated in the development of autoimmune disorders. For instance, the TAM system has been related to multiple sclerosis, an autoimmune demyelinating disease [60] and systemic lupus erythematosus (SLE), a chronic autoimmune disease that is associated to defective clearance of apoptotic cells [61]. Therefore, impaired TAM activity could trigger these disorders by promoting autoantigen exposure and reducing self-tolerance, leading to an uncontrolled immune response [14,62,63], as recently reviewed [64,65].

## 3. Functions in Coagulation and the Vasculature

**Figure 1:**
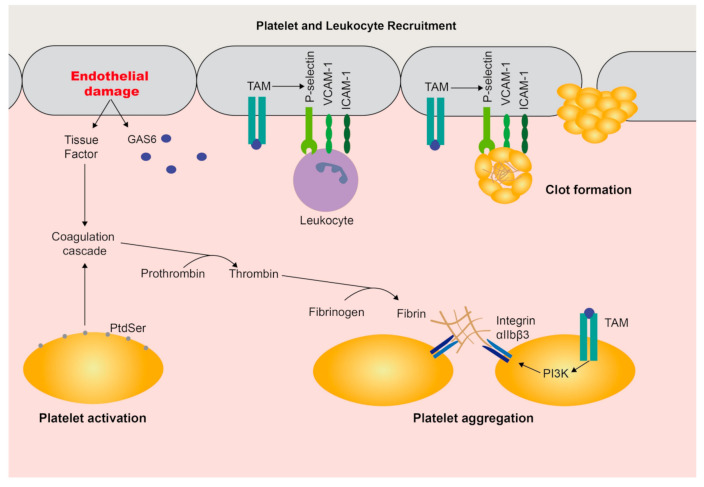
GAS6/TAMs in hemostasis. During damage, growth-arrest-specific 6 (GAS6) is secreted by endothelial cells and its signaling upregulates the expression of adhesion molecules (P-selectin, VCAM-1 and ICAM-1) in the endothelium (grey). Thus, platelets (yellow) and leukocytes (purple), such as neutrophils, are recruited to damaged tissue allowing their interaction with the vessel intima. Besides, activation of platelets leads to phosphatidylserine (PtdSer) exposure and interaction with vitamin K-dependent proteins (VKDPs), including TAM ligands. Pro-coagulant Gla-proteins trigger coagulation cascade activation, while TAM activation promotes platelet aggregation and stabilization of clots by phosphoinositide 3-kinase (PI3K)-dependent phosphorylation of β3 integrin increasing affinity to fibrinogen and other ligands.

After vascular damage, the platelets are activated to induce clot formation and repair the endothelium. Activated platelets expose PtdSer on the cell surface recruiting plasma VKDPs through their Gla-domain, including coagulation factors, PROS1, and GAS6 (Figure 1). Indeed, TAM signaling is involved in platelet aggregation and thrombus stabilization [66], as reviewed in [67]. In platelets, through the PI3K pathway, TAMs induce the phosphorylation of β3 integrin, thus amplifying integrin αIIbβ3 signaling and promoting aggregation [68]. Moreover, GAS6 is secreted by endothelial cells upon damage [69] and TAM activation increases the expression of adhesion molecules, such as ICAM-1 (intercellular adhesion molecule 1), VCAM-1 (vascular cell adhesion protein 1), or P-selectin promoting endothelial recruitment of platelets and leukocytes [70].

Therefore, GAS6 deficiency protects from thrombosis in mouse models due to defective endothelial activation and platelet aggregation [69,70,71] and higher circulating GAS6 levels have been associated to thromboembolism [72]. One has to bear in mind that PROS1 is an abundant VKDP in plasma, with important anticoagulant properties through its interaction with several components of the coagulation cascade in a TAM independent manner [73], a function that is not shared by GAS6 [74]. Further, PROS1 displays specific vascular effects that depend on its expression on vascular smooth muscle and endothelial cells, as demonstrated by cell specific ablation in mice [75].

Moreover, AXL activation contributes to vascular remodeling. GAS6/AXL promoted the survival and proliferation of vascular smooth muscle cells and endothelial cells, leading to the formation of new vessels [76,77]. It has also been described that AXL contributed to vascular endothelial growth factor A (VEGFA) signaling, which promotes the migration of endothelial cells and neovascularization [16]. AXL plays a critical role during vascular injury [78,79], and its angiogenic role has been widely explored in cancer and AXL inhibition successfully reduces angiogenesis and tumor growth in mouse models [76,80,81]. In models of vascular atherosclerotic damage and plaque progression, MERTK^+^ macrophages are critical for efferocytosis and prevention of necrotic plaques [82] and for the secretion of pro-resolving mediators such as TGF-β, IL-10 and resolvins [83]. In contrast, the GAS6/AXL axis seems to have a less important role in this setting [84,85], concomitant to a decrease in AXL gene and protein expression in advanced plaques [86].

All of these mechanisms have direct implications in cardiovascular diseases [85]. After heart failure, AXL expression is increased in heart tissue, while high blood levels of sAXL after receptor shedding predicted worse prognosis [87]. In contrast, MERTK expression in macrophages contributed to the efficient clearance of apoptotic cardiomyocytes promoting injury resolution after infarction in mice [88]. Overall, TAMs exert a complex role in the cardiovascular system comprising platelet aggregation, cardiovascular disease, and angiogenesis.

A clear example of the complex interaction of TAMs implicating the hemostatic and immune response to pathogens could be found in the progress of a COVID-19 illness. It has been observed that COVID-19 patients who had suffered lung failure and required mechanical ventilation, exhibited higher number of activated neutrophils and platelets in the circulation [89]. Neutrophils and platelets are known to activate each other, leading to the formation of obstructive blood clots in the lung and inducing immunothrombosis in COVID-19 [90]. Moreover, neutrophil extracellular traps (NETs) that normally trap and destroy bacterial and viral pathogens, seem to play an important role by stabilizing thrombi. Interestingly, recent data suggest that markers of NETosis and TAMs are simultaneously increased in critically ill COVID-19 patients [91]. Of note, while GAS6 and AXL are both augmented in the plasma of SARS-CoV-2 positive patients, GAS6 decreased in time in those patients that survived the infection [91]. Because of the possible contribution of GAS6 to neutrophil and platelet recruitment and aggregation, these observations may suggest GAS6/AXL as a promising target for the prevention and treatment of pulmonary failure and thrombotic complications of COVID-19. Moreover, it has been hypothesized that the dysregulation of blood coagulation might deplete circulating PROS1 levels and, consequently, contribute to the COVID-19-induced cytokine storm, thereby reducing the immunosuppressive action of MERTK in macrophages and diminishing PROS1 intrinsic anticoagulant function [58,92]. Pulmonary circulation could be especially dependent on natural anticoagulant mechanisms that are provided by endothelial synthesized proteins, such as PROS1. In this context, it is interesting that sMERTK is associated with pulmonary arterial hypertension in certain conditions [93]. The regulation of hemostasis and inflammation by TAM receptors and their ligands in the context of viral infections should be addressed in future research.

## 4. Viral Infection

**Figure 2:**
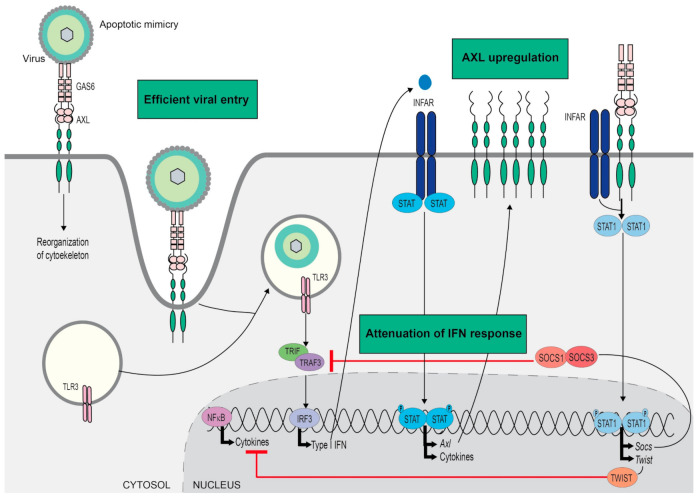
AXL implication in viral infection. Specific viruses take advantage of TAM role in efferocytosis bridging viral envelope phosphatidylserine (PtdSer) to target cells, a strategy named apoptotic mimicry. AXL intracellular signaling induces reorganization of cytoskeleton and promotes engulfment of virus increasing infectivity. Further, toll like receptor (TLR) stimulation by viral particle and the consequent type I interferon (IFN) antiviral response upregulate AXL expression. Then, AXL, interacting with IFN receptor (INFAR), switches off TLR and IFN inflammatory response via induction of suppressor of cytokine signaling proteins (SOCSs) and TWIST. GAS6, growth-arrest-specific 6; IRF, interferon-regulatory factor; NF-κB, nuclear factor-κB; STAT, signal transducer and activator of transcription; TRAF, tumor-necrosis-factor-receptor-associated factor; TRIF, Toll/interleukin-1-receptor-domain-containing adaptor protein inducing interferon-β.

Mimicry is a common strategy that is used by pathogens to increase their infectivity. For instance, PtdSer exposed at the virus envelope would interact with the phagocytic machinery of target cells, inducing its internalization [94]. The involvement of TAM receptors in viral apoptotic mimicry was first described by Morizono et al. in vaccinia viruses. GAS6 binding to virus PtdSer promoted efficient entry and the replication of virus [95]. TAM contribution to infection was later observed in several human pathogens such as Ebola, Dengue, West Nile virus and Zika [96,97,98], reviewed in [99]. A different mechanism was found in SV40, a non-enveloped polyomavirus, which interacts with TAMs directly by mimicking the ligand binding sites [100]. TAM inhibitors or mutated TAM receptors reduce viral infectivity, demonstrating that complete TAM signaling is required for potent virus infection [95,98] (Figure 2).

Moreover, TAM activation by enveloped virus moderated antiviral dendritic cell response reducing type I IFN signaling [101]. In fact, the small-molecule TAM inhibitor BMS-777607 demonstrated an antiviral effect during the lentivirus challenge of BMDCs by recovering the IFN signaling. BMS-777607 is also used as a c-MET inhibitor in human clinical trials; however, this effect was TAM-related as verified in TAM KO BMDCs. Although TAM targeting has been proposed as a therapeutic strategy in enveloped-virus infection [97,102], efferocytosis by APCs allows processing and presentation of antigens and TAM inhibition may impair the onset of an efficient adaptive immunity. Indeed, TAM deficiency reduced cross-presentation and T-cell cytotoxic activity, and *Axl*−/− mice showed higher virus load and lethality after herpes simplex virus-1 infection [103,104].

**Figure 3:**
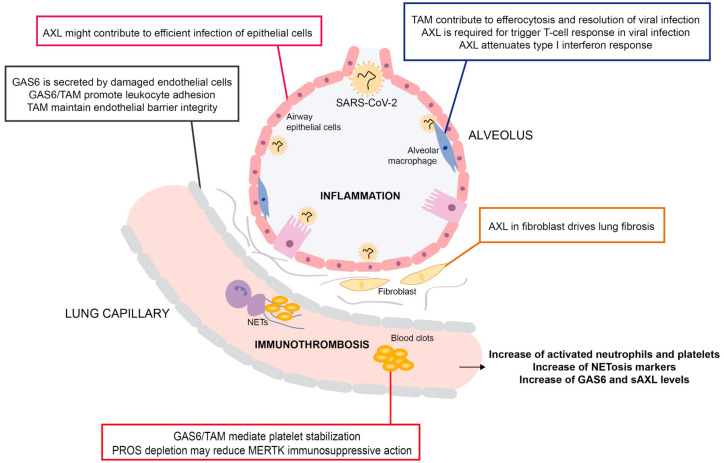
Processes implicated in COVID-19 role with a role of TAM receptors. TAM may have a role at different levels in COVID-19 pathogenesis. First, in airways, TAM might enhance infection promoting efficient virus entry to epithelial cells and inhibiting anti-viral interferon response. However, TAM could be required for stimulation of adaptive immunity and damage resolution. Of note, relating long-term complications, over-activation of AXL in lung fibroblast can be critical for pulmonary fibrosis development. Second, we suggest an important role of TAM signaling in coagulopathies linked to excessive inflammation observed in COVID-19. TAM regulation of vessel permeability, recruitment of neutrophils and stabilization of platelets may affect immunothrombosis, characterized by neutrophils and platelets activation, neutrophil extracellular traps (NETs) release and clot formation. Interestingly, COVID-19 patients present not only an increase of NETosis markers and activated neutrophils and platelets in blood, but also increased levels of GAS6 and sAXL. Furthermore, a decrease in PROS1 activity would unbalance hemostasis, increasing the risk of local immunothrombosis.

According to this view, GAS6 and TAMs would be expected to participate in COVID-19 viral entry (Figure 3). Actually, a preprint publication suggests an interaction of AXL with SARS-CoV2 promoting infection in epithelial cells [105]. In line with it, the AXL inhibitor gilteritinib, which is an FDA-approved drug for the treatment of acute myeloid leukemia, was recently demonstrated to possess antiviral efficacy against SARS-CoV-2 infection in Vero E6 cells, providing additional grounds for GAS6/AXL targeting as a promising anti-COVID-19 treatment [106]. Because gilteritinib is a tyrosine kinase inhibitor not only highly selective for both FLT3 and AXL, but also with weak activity against other kinases, such as c-KIT [107], non-selective AXL mechanisms should not be completely discarded. In this context, it is interesting to note that the AXL inhibitor Bemcentinib (BGB324), currently undergoing Phase II clinical trials for several cancer types, has been fast-tracked towards Phase II clinical trials by the UK government-funded ACCORD (Accelerating Covid-19 Research & Development platform) study, while other AXL inhibitors have been proposed as possible treatments for COVID-19 [106].

However, TAM receptors may play other roles during viral infection that may complicate TAM intervention and its translation into medical practice. In studies with the neuroinvasive West Nile and La Crosse encephalitis viruses, blood-brain barrier permeability depended on TAM presence, and the brains of *Mertk−/−* and *Axl−/−* mice exhibited enhanced virus entry and infection [108]. Similarly, MERTK regulated endothelial barrier integrity during inflammatory response in a mouse model of acute pneumonia [109]. Moreover, IFN signaling during viral infections induced AXL expression in lung macrophages to prevent excessive inflammation and clear any damaged cells [110,111]. Bacterial coinfections are another aspect to take into account, which have a profound effect on COVID-19 associated mortality [112]. A recent publication using a RSV mouse model demonstrated that AXL was crucial for attenuating the immune response to pneumococci, while GAS6/AXL blockade restored antibacterial protection [113]. From these data, the GAS6/AXL axis appears as a mechanism evolved to provide efficient clearance of respiratory viral infections, while adapting the subsequent immune response in order to avoid excessive organ damage and dysfunction (Figure 3). The risk of bacterial secondary infections is the price to pay in this tradeoff.

Therefore, the benefits and detriments of TAM inhibition for viral infection need to be considered in future studies. Besides bemcentinib, gilteritinib, and BMS-777607, other AXL inhibitors have already reached the clinic. Of note, most of them exhibit efficacy against other tyrosine kinases, such as cabozantinib, which is also a potent VEGFR2 and c-Met inhibitor [114] or merestinib with activity against MET and MST1R [115]. Moreover, MERTK or pan-TAM inhibitors, such as UNC2025 [116] or RXDX-106 [117], are also obtaining interesting results, increasing the collection of compounds, each one with different off-target effects that may be used in TAM research. Most likely, TAM modulators, depending on the timing of administration, may have prophylactic properties or alter adaptive immunity. Highlighting the importance of the treatment schedule, recent data revealed that GAS6 and AXL significantly increased in plasma of COVID-19 patients as compared to controls, while GAS6 decreased over time in patients surviving to 30 days post ICU admission. In any case, it is noteworthy that the TAM role on virus infection is undergoing an accelerated translation into clinical practice.

## 5. Conclusions

Over the last decade, TAM receptors and ligands have been strongly connected to different human pathologies [63,81,88,102,118,119]. The TAM system, first related to the immune system and cancer, and more recently in molecular processes ranging from induction of fibrosis to cytokine control, is now establishing new links to other diseases. Amid the flood of data generated in the face of the COVID-19 pandemic, reliable reports are emerging that support the important role of the TAM receptor family in SARS-CoV-2 infection and COVID-19 disease progression. Specific alterations in the serum levels of sAXL, sMERTK, GAS6, or PROS1 have been previously related to the progression of fibrosis and inflammatory diseases in liver, heart or lung. Alterations in certain components of the system in COVID-19 patients seem associated to disease severity and clinical complications. Therefore, COVID-19 can probably be added to the list of diseases that are potential beneficiaries of TAM targeting. Better knowledge of the mechanisms involved and well-designed preclinical studies will expedite the access of TAM modulators to medical trials and help patients not only for COVID-19, but also for other viral illnesses or with related coagulopathies with a strong implication of the immune response.
